# Objective Assessment of Fall Risk in Parkinson's Disease Using a Body-Fixed Sensor Worn for 3 Days

**DOI:** 10.1371/journal.pone.0096675

**Published:** 2014-05-06

**Authors:** Aner Weiss, Talia Herman, Nir Giladi, Jeffrey M. Hausdorff

**Affiliations:** 1 Laboratory for Gait & Neurodynamics, Movement Disorders Unit, Department of Neurology, Tel-Aviv Sourasky Medical Center, Tel-Aviv, Israel; 2 Department of Neurology, Sackler Faculty of Medicine, Tel-Aviv University, Tel-Aviv, Israel; 3 Sagol School of Neuroscience, Tel-Aviv University, Tel-Aviv, Israel; 4 Department of Medicine, Harvard Medical School, Boston, Massachusetts, United States of America; 5 Department of Physical Therapy, Sackler Faculty of Medicine, Tel-Aviv University, Tel-Aviv, Israel; Cardiff University, United Kingdom

## Abstract

**Background:**

Patients with Parkinson's disease (PD) suffer from a high fall risk. Previous approaches for evaluating fall risk are based on self-report or testing at a given time point and may, therefore, be insufficient to optimally capture fall risk. We tested, for the first time, whether metrics derived from 3 day continuous recordings are associated with fall risk in PD.

**Methods and Materials:**

107 patients (Hoehn & Yahr Stage: 2.6±0.7) wore a small, body-fixed sensor (3D accelerometer) on lower back for 3 days. Walking quantity (e.g., steps per 3-days) and quality (e.g., frequency-derived measures of gait variability) were determined. Subjects were classified as fallers or non-fallers based on fall history. Subjects were also followed for one year to evaluate predictors of the transition from non-faller to faller.

**Results:**

The 3 day acceleration derived measures were significantly different in fallers and non-fallers and were significantly correlated with previously validated measures of fall risk. Walking quantity was similar in the two groups. In contrast, the fallers walked with higher step-to-step variability, e.g., anterior-posterior width of the dominant frequency was larger (p = 0.012) in the fallers (0.78±0.17 Hz) compared to the non-fallers (0.71±0.07 Hz). Among subjects who reported no falls in the year prior to testing, sensor-derived measures predicted the time to first fall (p = 0.0034), whereas many traditional measures did not. Cox regression analysis showed that anterior-posterior width was significantly (p = 0.0039) associated with time to fall during the follow-up period, even after adjusting for traditional measures.

**Conclusions/Significance:**

These findings indicate that a body-fixed sensor worn continuously can evaluate fall risk in PD. This sensor-based approach was able to identify transition from non-faller to faller, whereas many traditional metrics were not successful. This approach may facilitate earlier detection of fall risk and may in the future, help reduce high costs associated with falls.

## Introduction

Falls are a debilitating problem among patients with Parkinson's disease (PD), leading to diminished mobility, poor quality of life, morbidity, and mortality [1], [2]. About 50–70% of patients with PD fall at least once every year [1]–[3], however, tools for identifying this sub-group are not yet optimal [1], [4]. Assessment of fall risk in PD generally relies on self-report and performance-based tests [1], [4]–[7]. While informative, these approaches suffer, to varying degrees, from recall bias and poor sensitivity, reflect the performance at a single point in time, and may be influenced by the reverse “white coat syndrome”, where patients walk relatively well when they are examined by a physician.

Falls in PD are associated with stress, executive function, dopaminergic and cholingeric function, dyskinesia, motor response fluctuations and freezing of gait [1], [4], [5], [8]–[12]. These phenomena fluctuate during the day and likely change fall risk. Metrics based on the evaluation of the risk of falls of patients with PD over a relatively extended time period, as subjects carry out their routine activities of daily living in their home and community environment may be one of the keys to improving the reliability and sensitivity of the assessment of fall risk. These metrics could potentially allow for earlier intervention and, ultimately, lower the high costs that result from falls. This possibility is also responsive to the more general eHealth and mobile Health (mHealth) initiatives for remote, at-home assessment of health and function [13], [14].

The objective of the present study was to determine whether metrics derived from a small, body-fixed sensor (i.e., an accelerometer) worn continuously for 3 days, are able to capture fall risk among patients with PD. In a pilot study [15] among community living older adults (i.e., people without PD), acceleration measures differed between fallers and non-fallers. However, it is not clear if this approach can be applied to identify fall risk among patients with PD. Therefore, here, we aimed to: 1) test if sensor-derived metrics differ in PD fallers and PD non-fallers, based on self-report of fall history; 2) assess whether these metrics are associated with performance-based tests of fall risk; and 3) evaluate whether sensor-derived measures are able to assist in the estimation of the time to first fall among subjects who reported no falls in the past.

## Materials and Methods

### Subjects

110 patients with PD participated in a cross sectional study focusing on PD motor subtypes [16]. They were recruited from the outpatient Movement Disorders Unit at the Tel-Aviv Medical Center and from other affiliated clinics around the country. Data from 107 patients were analyzed in the present study. Subjects were included if they were diagnosed by a movement disorders specialist with idiopathic PD (as defined by the UK Brain Bank criteria), were between 40 and 85 years of age, had a Hoehn & Yahr score between I and IV, if they were ambulatory, and if they had a Mini Mental State Examination (MMSE) score above 24 points. Subjects were excluded if they have had brain surgery or had significant co-morbidities likely to affect gait, e.g., acute illness, orthopedic disease, or history of stroke. Subjects who could not walk in the OFF medication cycle and subjects who could not comply with the protocol were excluded.

### Ethics

Ethics approval from the human studies committee of the Tel Aviv Sourasky Medical Center was obtained and all participants provided informed written consent, according to the Declaration of Helsinki.

### Clinical assessment

Parkinsonian symptoms, disease duration, and disease severity were assessed based on interview and the Unified Parkinson's Disease Rating Scale (MDS-UPDRS) [17]. Fall history in the year prior to testing was assessed based on self-report. Assessment of falls in the one year following baseline testing was evaluated via fall calendars completed by the subjects and returned once a month, following recommended procedures [18]. As previously suggested, a fall was defined as “Unintentionally coming to rest on the ground, floor, or other lower level” [18], [19]. The MMSE was used as a general measure of cognitive function. The new freezing of gait questionnaire (NFOG-Q) evaluated if the subject experienced freezing episodes and the severity of this symptom [20]. Gait speed was determined by measuring the average time the subject walked the middle 10 meters of the lab corridor at their comfortable pace. In addition to fall history, a known predictor of future falls, other “traditional” measures of fall risk included performance-based measures of balance and mobility: the pull test [17], Dynamic Gait Index [21], the Berg Balance Test [22], and the Timed Up and Go [23]. The Geriatric Depression Scale [24] and the Activity-specific Balance Confidence Scale [25] assessed depressive symptoms and balance confidence, respectively.

### 3 day assessment of gait and mobility

After undergoing the clinical assessment, patients wore the sensor on their lower back for 3 consecutive days (except during activities such as showering). Subjects received a diary to track when and why they took off and put on the device [15]. The data acquisition device and signal processing were previously described in a pilot study [15]. In that study, an approach for quantifying mobility under non-stationary conditions [26] was applied to assess fall risk among community dwelling, (non-PD) older adults [15]. Briefly, participants wore a small, light-weight sensor (McRoberts, DynaPort Hybrid system, The Netherlands) on a belt on the lower back. This location, although centered, has previously shown to reflect lower extremity movement during walking [27]. The units' dimensions are 87×45×14 mm (74 grams). The Hybrid includes a triaxial accelerometer (sensor range and resolution are: ±2g and ±1mg, respectively) and a triaxial gyroscope (data not analyzed in the present study). The 3 acceleration axes studied were: vertical, medio-lateral, and anterior posterior. Data was saved on an SD card at 100 Hz, and later transferred to a personal computer for further analysis (using Matlab, the Mathworks software).

The data analysis of the 3 day recordings included two stages [15], [26]: 1) Detection of all walking segments, from which only the bouts with duration of above one minute were selected; and 2) Application of acceleration derived measures to the walking segments that were identified in the previous stage. We note that this approach was used to ensure that the analyses were based on relatively long walking episodes which is important for assessing walking quality. We recognize, however, that in terms of quantifying the total number of steps and time spent walking, this approach underestimates the total time spent walking throughout the day since short walking episodes are not included in the analyses. In the first stage, a filter was applied to extract the walking segments of the 3-day recordings (adapted from previous work [26]). The algorithms are able to detect even walking performed at relatively low speeds, as they are based on the combination of two detection methods [15], [26]. Metrics that reflect the quantity and quality of the walking activity were determined in the second stage. Quantity measures included the total number of walking bouts, the percent of time spent walking which reflects the total walking in relation to the overall walking and non-walking activity, the total number of steps, median walking bout duration, median number of steps, and median cadence per bout. Quality related sensor-derived measures included: frequency-derived measures that reflect variability of the gait pattern [26], regularity measures that reflect gait rhythmicity and consistency [28] and the harmonic ratio which is an index of gait smoothness [29]. Construct and concurrent validity has been established previously for many of these measures [15], [26], [28]–[31]. In addition, we applied step-to-step analyses to evaluate the Phase Coordination Index (PCI) [32], which is a measure of the consistency and accuracy of the left-right bilateral coordination during walking, i.e., timing of one foot with respect to the other. A fatigue index was calculated for these measures as the percentage of difference between the gait measures of the first and last 30 second intervals of each activity bout.

### Statistical analysis

Statistical analyses were performed using SPSS version 21. Subjects were classified as fallers if they reported at least 1 fall in year prior to baseline testing. Normality was assessed using the Kolmogorov-Smirnov test. Based on this check, either Student's t-tests or the Mann-Whitney test was used to evaluate our first aim and compare the fallers and non-fallers. To test our second aim, Spearman's correlations were performed to assess the associations between the different clinical tests of fall risk and measures derived from the 3 day recordings; Spearman's correlations were used as a more conservative estimate compared to Pearson's; although in general the results were similar. To test our third aim, survival analyses using Kaplan-Meier tests assessed if acceleration measures were associated with time to first and time to second fall (i.e., transition to multiple faller) among the subjects who reported no falls in the past. In addition, Cox regression analyses were applied to evaluate if the accelerometer derived measures were associated with falls during the follow-up period when adjusting for more traditional clinical measures. Group values were reported as mean±standard deviation. Corrections for multiple comparisons were made using the Hochberg-Benjamini method [33]. This was done for each axis separately since the anterior-posterior, vertical and medial-lateral directions likely represent different locomotor constructs [15], [15], [34]–[38].

## Results

Subject characteristics are shown in Table 1. Three subjects were excluded due to technical problems (device failure or loss). Thus, 107 patients were included in the analysis. Two patients had less than one day recording (<16 h), and four patients had approximately two days of data (43–52 h) due to SD card initialization error or early device removal. Most of the quantity measures were not affected by the recording duration since they were normalized as percentage from the overall activity or from the activity bouts. The remaining patients had in average 73.5±2.9 hours of recording.

**Table 1:** Demographics, functional performance, and fall risk measures in fallers and non-fallers.

	PD Fallers	PD Non-Fallers		P-value
**Demographics and disease related measures**				
# of subjects	40	67		-
Age (yrs)	66.50±8.21	64.00±9.76		0.178
Gender (% women)	35.00%	19.40%		0.072
Height (m)	1.67±0.08	1.70±0.08		0.042
Education (yrs)	16.15±3.66	15.19±3.76		0.294 †
Weight (kg)	79.17±14.75	76.29±10.41		0.283
Body-mass-index (kg/m2)	28.19±4.05	25.69±4.31		0.004 *
Levodopa equivalent dosage (mg/d)	400.10±353.61	454.63±341.81		0.432
Geriatric Depression Scale	3.75±3.21	4.21±3.48		0.721 †
Mini Mental Status Exam	28.99±1.17	28.38±2.18		0.367 †
Disease duration (years)	6.08±4.02	5.15±3.08		0.323 †
Hoehn & Yahr Stage §	1.5 (0)	1.5 (2)		0.007 † *
	2 (12)	2 (32)		
	2.5 (9)	2.5 (22)		
	3 (4)	3 (8)		
	3.5 (5)	3.5 (0)		
	4 (10)	4 (3)		
UPDRS motor score “ON”	33.44±11.41	33.93±12.60		0.842
UPDRS motor score “OFF”	40.78±13.07	40.15±13.35		0.814
New freezing of gait questionnaire	7.63±10.01	1.97±5.16		0.0003 † *
**Gait and fall risk measures**				
# of falls in the past year	5.85±16.01		0.0±0.0	<0.0001 † *
Dynamic Gait Index	20.70±3.36		22.54±1.44	0.0002 † *
Berg Balance Scale	49.83±7.81		53.43±3.16	0.001 † *
Timed Up and Go “OFF” (sec)	13.18±12.77		9.68±2.37	0.004 † *
Pull Test	1.43±1.29		0.93±1.17	0.047 †
Activities-specific Balance Confidence (%)	77.69±17.41		90.19±13.46	<0.0001 † *
Gait speed “OFF” (meter/sec)	1.01±0.23		1.18±0.16	<0.0001 *

† Measures which were not distributed normally according to the Kolmogorov-Smirnov test and therefore were analyzed with the Mann-Whitney test.

* Measures of functional performance that were significantly different in the two groups. According to the Hochberg-Benjamini method for multiple comparison analysis, a p-value less than or equal to 0.004 is considered statistically significant in the present analysis.

§ Each row represents a Hoehn & Yahr Stage and in brackets the number of patients in that stage

The cohort was stratified into fallers (n = 40) and non-fallers (n = 67). They did not differ with respect to age, gender, years of education, MMSE scores, levodopa equivalent dose, or weight. As expected, the fallers scored significantly worse on traditional measures of fall risk e.g., the Dynamic Gait Index and the Berg Balance Test. The fallers also walked more slowly and had greater levels of fear of falling as assessed by the Activities-specific Balance Confidence scale.

### 3 day measures and fall history

**Figure 1:**
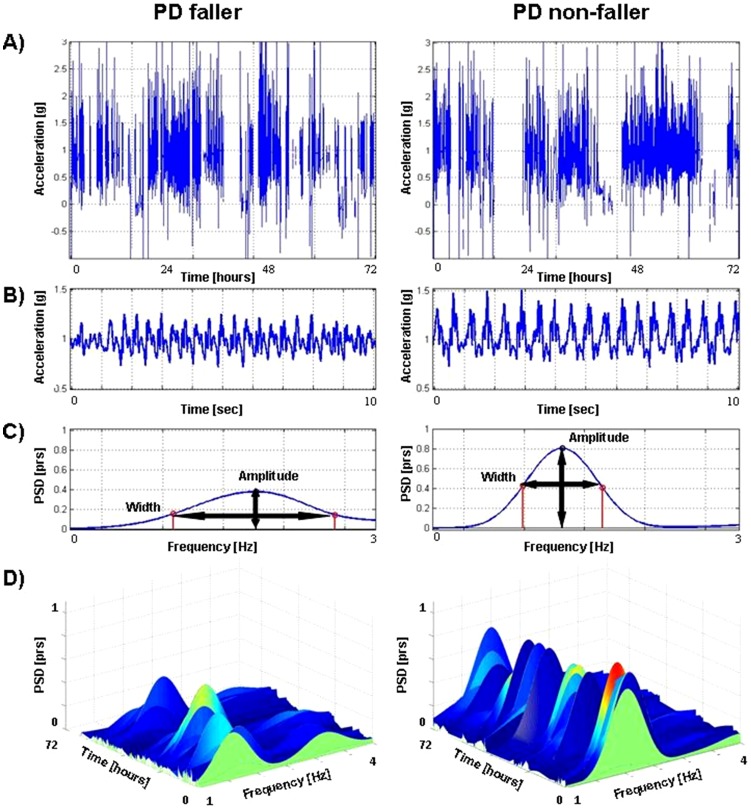
Examples of vertical acceleration signals of a PD faller (left) and a non-faller (right). Figure 1A shows a 3-day raw acceleration signal. Figure 1B and 1C show the time and frequency domains of a 30 second signal (derived from the raw signal), respectively. The acceleration pattern of the PD faller (male, 61 yrs old) is less smooth compared to the PD non-faller (male, 74 yrs old) (Figure 1B). The peak amplitude of the dominant frequency is lower and wider in the faller compared to the non-faller, indicating of a less consistent, more variable gait pattern (figure 1C). Figure 1D shows an example of 3-day vertical acceleration signal in the frequency domains. The PD faller has a less consistent gait pattern, as reflected by the lower amplitude and wider spectrum. Similar findings are observed on a group level (recall Table 3).

An example of a raw accelerometer signal obtained over the 3 days in a faller and non-faller is shown in Figure 1A. Figures 1B and 1C show a 30 second portion of this signal. The signal is smoother and more consistent in the non-faller. This can be seen in the time domain (Figure 1B) and in the frequency domain (Figure 1C), where the peak amplitude is larger and narrower in the non-faller. A similar picture is seen when we examine the changes in the power spectrum over the 3 day recording in a faller and non-faller (Figure 1D). The peaks are shorter and broader in the faller, reflecting a more variable and less consistent walking pattern. The results shown in Figure 1 were also seen on the group level, as detailed further below.

**Figure 2:**
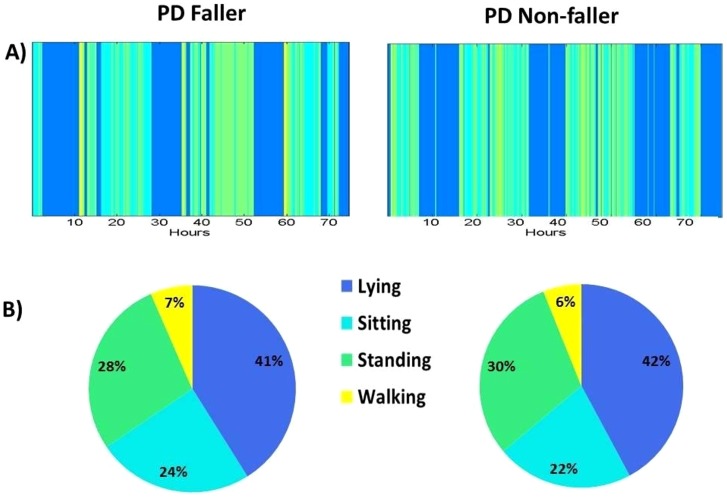
Time spent in different activities in a PD faller and non-faller (right). Figure 2A shows a general, descriptive example of the time spent walking, standing, lying and sitting in two subjects as a function of time over the 72 hour recordings. Figure 2B shows the percent time spent in each of these activities. Note that on a group level, walking amounts were similar in the fallers and non-fallers (see also Table 2). Please note that although this figure is based on previously validated measures [57], we do not extract any quantitative measures from it. The figure is included here to illustrate how the present approach can be extended further in future work. In the current study, the analyses focused on walking bouts that were one minute or longer in order to robustly identify walking and, ultimately, the quality of these walking bouts (recall the methods and Table 2).

Quantity of walking over the 3 days was similar in the fallers and non-fallers (see Table 2). Figure 2 graphically illustrates the idea that the quantity of walking was similar in the two groups. In contrast, measures related to the quality of gait differed in the two groups (see Table 3 and recall Figure 1C and 1D). For example, fallers had higher gait variability in the vertical and anterior posterior directions, as exhibited by a larger width of the power spectral density (p≤0.028). The fallers also had less consistency in the vertical direction, as depicted by the lower stride regularity (p = 0.018). A less smooth gait pattern was observed in the anterior-posterior and vertical directions, as exhibited by the lower vertical and anterior posterior harmonic ratio (p≤0.043) which is related to less gait smoothness [39]. The Phase Coordination Index was also significantly higher (i.e., worse) in the fallers compared to the non-fallers (fallers 8.30±8.62, non-fallers 6.68±8.29; p = 0.045).

**Table 2:** Acceleration derived **3-day** measures of the *quantity* of walking in the fallers and non-fallers.

Measure	PD Fallers	PD Non-Fallers	P-Value *
Quantity measures (Activity count)			
Total Number of activity Bouts [#] a	29.73±22.78	31.88±22.53	0.635
Total percent of activity duration [%]	2.18±2.04	2.25±1.94	0.597 †
Total Number of steps for 3-days [#] a	9392.22±9290.97	10658.82±9770.78	0.249 †
Median activity Bout duration [sec]	114.44±34.23	113.15±34.62	0.852
Median Number of steps for Bout [#]	190.30±61.77	198.94±64.29	0.496
Cadence [steps/minute]	103.70±16.21	108.37±10.37	0.109

These measures were calculated only from activity bouts ≥1 min, from the AP axis

* The measures in this table were not statistically different in the fallers and non-fallers.

† Measures which were not distributed normally according to the Kolmogorov-Smirnov test and were, therefore, analyzed using the Mann-Whitney test.

a Measures which were not normalized to the entire recording duration or activity duration.

Note: None of these measures were significantly different in the fallers and non-fallers.

**Table 3:** Acceleration derived **3-day** measures of the *quality* of walking in the fallers and non-fallers.

Measure	Axis	PD Fallers	PD Non-Fallers	P-Value
Amplitude of dominant frequency [prs]	V	0.57±0.19	0.67±0.18	0.012 *
	AP	0.57±0.17	0.59±0.13	0.605
	ML	0.19±0.15	0.17±0.15	0.285 †
Width of dominant frequency [Hz]	V	0.77±0.15	0.72±0.13	0.028 † *
	AP	0.78±0.17	0.71±0.07	0.012 † *
	ML	0.97±0.17	0. 91±0.12	0.033
Stride Regularity [g∧2]	V	0.48±0.12	0.55±0.14	0.018 *
	AP	0.50±0.10	0.54±0.10	0.063
	ML	0.35±0.11	0.39±0.13	0.128
Harmonic Ratio	V	2.01±0.48	2.23±0.54	0.043 *
	AP	1.84±0.48	2.09±0.48	0.011 *
	ML	0.65±0.15	0.59±0.13	0.035

* Measures which were significantly different in the fallers and non-fallers. We performed the Hochberg-Benjamini method for multiple comparison analysis for each of the 3 locomotor constructs separately: vertical (V), anterior posterior (AP), and medio-lateral (ML). P-values less than or equal to 0.043 (V), 0.012 (AP) and 0.00 (ML) were considered statistically significant in the 3 different constructs in the present analyses.

† Measures which were not distributed normally according to the Kolmogorov-Smirnov test and were, therefore, analyzed using the Mann-Whitney test.

Interestingly, when looking at the fatigue index, it was evident that while the non-fallers tended to increase their vertical stride regularity during a walking bout. The fallers tended to decrease their stride regularity (i.e. gait consistency worsened). The fatigue index of stride regularity was −4.85±15.91% in the fallers and 1.21±11.47 in the non-fallers (p = 0.009).

### Correlations between the 3 day measures and clinical measures of fall risk

Table 4 shows examples of the associations between the 3 day sensor-derived measures and performance-based measures of fall risk. Mild to moderate correlations were observed. The level of fear of falling was also significantly correlated with many of the gait quality measures (e.g., with vertical amplitude r = 0.31; p = 0.001). No significant correlations were found between the UPDRS motor score and the acceleration derived gait measures (r<0.138, p>0.068).

**Table 4:** Associations between the 3 day sensor-derived measures and performance-based measures of fall risk.

Measure		Axis	Gait Speed	Berg Balance Test	Dynamic Gait Index	Timed Up & Go
Amplitude of dominant frequency [psd]		V	0.40 (0.00001)	0.23 (0.016)	0.24 (0.011)	−0.35 (0.0001)
		AP	−0.01 (0.881)	0.07 (0.433)	0.07 (0.465)	0.03 (0.737)
		ML	−0.33 (0.0004)	−0.29 (0.002)	−0.13 (0.182)	0.28 (0.003)
Width of dominant frequency [Hz]	V		−0.23 (0.016)	−0.23 (0.015)	−0.101 (0.305)	0.23 (0.017)
	AP		−0.36 (0.0001)	−0.30 (0.002)	−0.25 (0.009)	0.32 (0.001)
	ML		−0.06 (0.529)	0.06 (0.511)	−0.003 (0.972)	−0.006 (0.954)
Harmonic Ratio	AP		0.39 (0.0002)	0.34 (0.0003)	0.34 (0.0003)	−0.31 (0.001)

The p-values were corrected for multiple comparisons according to the Hochberg-Benjamini method. We performed the correction for each of the 3 locomotor constructs separately: vertical (V), anterior posterior (AP), and medio-lateral (ML). P-values less than or equal to 0.017 (V), 0.015 (AP) and 0.003 (ML) were considered statistically significant in the 3 different constructs respectively in the present analyses.

### Survival Analysis

**Figure 3:**
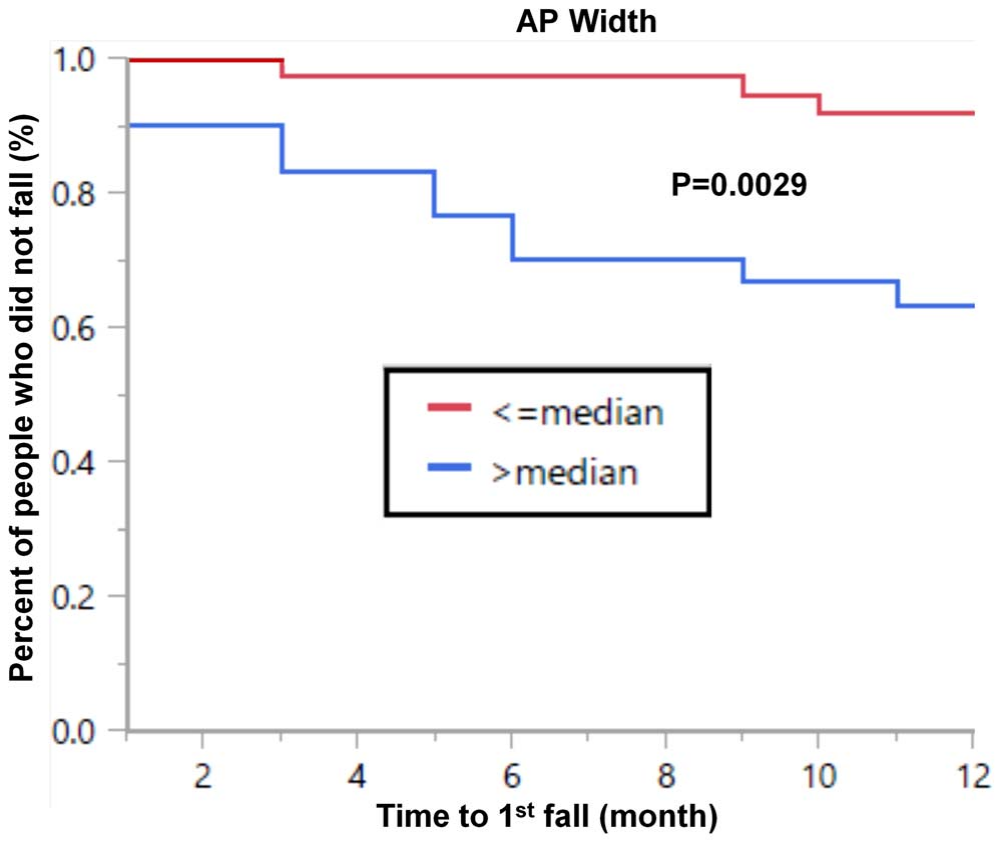
Survival curve showing the time to first fall among all subjects who reported no falls in the year prior to the study. Based on fall history, all of these subjects had a relatively low risk of future falls. However, the anterior-posterior width of the peak in the frequency domain, a measure of gait variability derived from the 3-day recording, was associated with time to first fall. When subjects were classified as those having a relatively high (above the median) or low (below the median) width, those with a high width experienced a fall sooner (Log rank test: p = 0.0034, Wilcoxon test: p = 0.0029), compared to those with a relatively low width.

**Figure 4:**
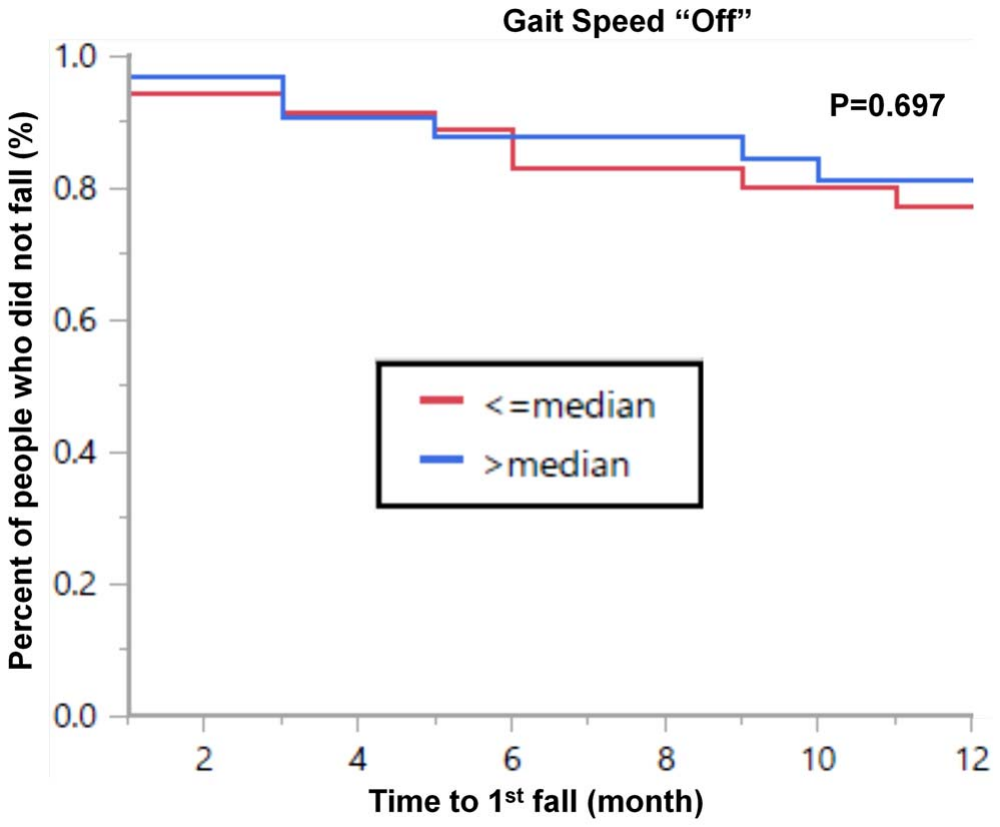
Survival curve showing that gait speed while off was not significantly associated with the time to first fall (Log rank test: p = 0.688, Wilcoxon test: p = 0.697) among subjects who reported no falls in the year prior to testing. Please compare to Figure 3.

*A*mong all subjects, several acceleration measures were associated with future fall status. For example, survival analysis for the entire cohort showed that time to first fall in the year following the study occurred significantly sooner in subjects with a more variable, less consistent gait pattern, based on the 3 day measures (e.g., anterior-posterior width: Log rank test: p = 0.0018, Wilcoxon test: p = 0.0014). History of falls is a known, strong predictor of future falls [4]; we confirmed this in our study as well (survival analysis for the entire cohort showed that time to first fall in the year following the study occurred significantly sooner in subjects with fall history: Log rank test: p<0.0001, Wilcoxon test: p<0.0001). Therefore, to evaluate the possibility of identifying “new” fallers, we conducted an analysis that focused on subjects who reported no falls in the year prior to baseline. The anterior-posterior width was successfully able to identify time to first fall. Survival analysis among the subjects who reported no falls in the year prior to baseline (N = 67) demonstrated that the change in status from non-faller to faller (which occurred in 14 patients) occurred significantly sooner in subjects whose 3 day gait pattern was more variable on a stride-to-stride basis (i.e., an anterior-posterior width above the median), compared to those with a less variable pattern (i.e., below the median). This was true for time to first fall (Log rank test: p = 0.0034, Wilcoxon test: p = 0.0029) (see Figure 3). A trend was also seen with respect to the time to second fall (Log rank test: p = 0.120, Wilcoxon test: p = 0.106) (data not shown). When repeating the survival analysis for traditional measures of fall risk, only the Berg Balance Scale was significantly associated with time to first fall (Log rank test: p = 0.022, Wilcoxon test: p = 0.025). Other traditional measures were not significantly associated with time to first fall among subjects who reported no falls in the year prior to testing, e.g., Timed Up and Go while OFF (Log rank test: p = 0.196, Wilcoxon test: p = 0.185), the Dynamic Gait Index (Log rank test: p = 0.895, Wilcoxon test: p = 0.885), NFOG-Q (Log rank test: p = 0.118, Wilcoxon test: p = 0.143), gait speed while OFF (Log rank test: p = 0.688, Wilcoxon test: p = 0.697) (see figure 4), UPDRS motor score while OFF (Log rank test: p = 0.438, Wilcoxon test: p = 0.447), and disease duration (Log rank test: p = 0.069, Wilcoxon test: p = 0.077).

We also performed a Cox multiple regression analysis for the anterior-posterior width, adjusting for age, gender, disease duration, freezing of gait (i.e., NFOG-Q), Berg Balance Scale, gait speed and Timed Up and Go while OFF, Dynamic Gait Index and UPDRS motor score while OFF. The anterior-posterior width was still significantly associated with time to 1^st^ fall during the 12 months of follow-up, even after adjusting for all the covariates (p = 0.0039). The only covariate that remained significant in the model was the disease duration (p = 0.0009). The risk ratio for experiencing a fall in any month during the follow-up period was 7.03 with a 95% confidence interval (1.82–37.13). In other words, patients with a larger anterior-posterior width had a seven fold higher risk of experiencing a fall compared to those with a lower anterior-posterior width.

### Correlations among the derived measures

Table S1 shows the correlation among the sensor derived measures. The descriptive properties including distributions are shown in Table S2.

## Discussion

The present findings confirm that traditional measures of fall risk, i.e., fall history, gait, freezing of gait, and reduced performance on functional-performance tests of mobility, are associated with fall risk in patients with PD [1], [4]–[7]. The results also indicate that new measures based on the 3 day sensor-derived recordings reflect fall risk in patients with PD and that these metrics have the potential to enhance the evaluation of fall risk. Three lines of evidence support this idea: 1) When subjects were classified as fallers and non-fallers based on their fall history, several 3 day derived measures differed in the two groups. 2) Scores on traditional performance-based tests of fall risk were associated with the measures that were extracted from the 3 day recordings. 3) A 3 day measure predicted time to first fall among the subjects who reported no falls in the year prior to the baseline testing, whereas many traditional measures did not. These results all support the validity of using the 3 day measures in patients with PD and demonstrate the potential utility and value of this approach.

### Quantity versus Quality

The distinction between quality of gait versus quantity has been previously discussed [15], [26], . Interestingly, the amount of activity (i.e., quantity) during the 3 days of daily-living was similar in the PD fallers and non-fallers (recall Table 2), while the gait quality was different (recall Table 3). PD fallers walked with increased gait variability, a less consistent gait pattern, and less smooth gait. These group differences are consistent with previous studies conducted in laboratory settings (i.e., straight line, standardized walking) [8], [12], [39], [42]. Here, we extend those findings and demonstrate that the PD fallers and non-fallers differ in their gait quality when evaluated in everyday settings which likely involve more complex activities than in the clinic or lab. PD fallers apparently have a reduced ability to properly regulate gait in their natural environment, perhaps due to increased postural instability, loss of inter segmental flexibility, and/or deficits in rhythmicity [12]. While fall history is a well-known simple marker of future falls (in older adults and PD) [1], [2], [4], we show here, for the first time, that a sensor-derived metric that reflects gait quality can predict the time to a first fall and future fall status, even among subjects who reported no falls in the year prior to testing (recall Figure 3). This provides further support for the potential contribution of the 3 day derived measures; they can identify fall risk even before the first fall occurs.

The relationship between activity and fall risk is rather complex [43]. On the one hand, people who are more active may be more prone to fall due to their exposure to more hazardous situations. On the other hand, they may have a lower fall risk since they are healthier and more fit compared to less active people. Among community living older adults, there is support for both of these associations and the full explanation may be dependent on the nature of the activity and the fall [44]. In the present study, activity in the PD fallers and non-fallers were not statistically different, perhaps due to the wide range observed in both groups (recall Table 2). Although activity amounts have previously been related to the progression of PD [45], [46], the amount of walking activity does not seem to be a key to fall risk in patients with PD, at least among those with mild to moderate disease severity. Further studies should examine this question. Regardless of the relationship to fall risk, metrics derived from the body-worn sensor enable the accurate assessment of activity, as reported recently [45], [46], and can be used clinically to monitor and promote physical activity, providing details about the amount and distribution of common activities (recall Figure 2).

### Parkinson's disease fallers versus Older Adult Fallers

In a recent pilot, we assessed fall risk in healthy older adults [15] using the approach based on body fixed sensors taken in the present study. Here we extend that approach to the PD population, representing a neurodegenerative disease. It may be informative to consider the present results in relation to those reported in the previous study. Note that the cohort form the pilot study was about 12 years older than the PD patients in this study, so the results are not directly comparable. Still, both the older adult fallers and the PD fallers walked with increased variability in the anterior-posterior and vertical directions, compared to their non-faller controls. This provides further support for the utility of these measures and their ability to assess fall risk, even among a group of patients who all suffer from PD. Older adult fallers walked with *reduced* medio-lateral variability compared to healthy older adults [15]. This is consistent with the idea that higher medio-lateral variability is healthier and that a *reduced* ability to adapt to changing environmental conditions is reflected in lower medio-lateral variability [37]. In contrast, in PD, there was a tendency for the fallers to walk with *increased* medio-lateral variability compared to non-fallers (although not statistically significant), perhaps a reflection of poor axial control. Further investigation should be under taken to understand the apparently unique underlying construct of medio-lateral variability in patients with PD and the degree to which it differs from that of PD-free older adults.

### Potential Clinical Utility and Impact

Previously reported methods for assessing fall risk in patients with PD have merit [1], [4]–[7]. They do better than chance, can be applied in the clinical setting, and some have even been applied in the home. However, falls place a tremendous burden on patients and their families, severely restricting quality of life and functional independence. In most Western countries, 1–2% of all healthcare expenditures are spent on costs related to falls. Even a small reduction in fall frequency can lead to significant economic savings [46] and improvement in function. Improvement in the evaluation of fall risk should, therefore, lead to an alleviation of some of the burden of falls in PD.

Several recent studies have used body-worn sensors and continuous monitoring to study activity amounts in PD, the effects of deep brain stimulation in PD, and dynamics of physical activity in other cohorts [45]–[49]. As far as we know, our study is the first to use this type of approach to quantitatively evaluate fall risk in PD based on actual performance in the home and community setting. The results support the validity and potential of this low cost, easy-to-use technology. The sensors apparently can be used for early detection of fall risk (recall Figure 3). The results suggest that, in the future, perhaps this type of an approach can lead to economic savings and improved healthcare. In a sense, then, the study directly addresses an unmet clinical need.

A typical neurologist and geriatrician only has a few, rushed minutes to conduct a motor examination and to assess the risk of falls, one of the most important consequences of impaired motor function. This situation is the norm for the clinical assessment of patients with PD, for many patients with neurological disease who have a high risk of falls (e.g., patients post-stroke, individuals with multiple sclerosis, or Alzheimer's disease), and for older adults more generally. Body-worn sensors have the potential to improve the clinical assessment. Imagine that a few weeks before a clinical exam is scheduled, the patient receives in the mail a small, light-weight, body-worn sensor, the size and weight of a small sticker. This comes with simple instructions that explain how to wear the ready-to-use device that is worn like a small patch, and how to return the device (or the data) after it is worn for an extended period of time (e.g., 3, 7 or even 30 days). The data can be easily delivered to the clinician via post or the internet. The clinician then receives a detailed report of the patient's motor function (e.g., how many steps they walk per day, how much time they spend being active, lying down, or sitting; as in Figure 2) and fall risk (with a summary fall risk index and additional details for therapeutic targeting and tracking). All of this information can be made available to the clinician before the patient even enters the clinic. Equipped with this information and a summary report of fall risk and activity, the clinician can conduct the clinical examination informed with details about the patient's motor abilities, performance, and risk of falls. Perhaps, it is time to start to think about a clinical exam that is enhanced by objective, reliable and sensitive measures based on long-term recordings.

### Limitations and future work

Clinometric properties like the minimally clinical significant difference have not been fully established yet and further investigations are needed to pave the way for this type of clinical use of body-worn sensors. One might argue that many of the gait measures are inter-related and may be influenced by speed. However, several gait parameters clearly reflect independent constructs that are largely independent of each other [42], [50]–[56]. One might also argue that a more conservative approach to the multiple comparisons than the one we used, such as Bonferroni correction, would not have yielded statistically significant results. However, the Hochberg Benjamin method has been widely used and cited thousands of times since 1995 [33]. It was developed to address the relatively conservative Bonferroni correction, which may alter the interpretation of the results by considering the worst case only. The present analyses were based on 3 day recordings. In the future, it may be useful to compare between activities of day 1, 2 or 3 and to evaluate the trade-offs of using longer or shorter recording periods. It may also be helpful to derive other metrics from these signals to tease out the role of the dopaminergic and cholinergic systems in fall risk in PD, for example, and to provide insight into additional clinical aspects of PD symptoms and disease progression. Follow-up studies in larger cohorts will also be helpful to confirm the present results and more fully evaluate clinical utility and other clinometric properties.

In summary, these initial findings suggest that a body-fixed sensor worn for 3 days can be used to evaluate fall risk in patients with PD as they carry out activities in their natural home and community settings. The study cohort is fairly representative of the general PD population as there was a relatively broad range of motor function and disease duration (recall Table 1 and Herman et al. [16]) and as noted above, generalizability is supported by the consistency of the present results with the previous pilot study. This approach may improve our knowledge of the patient's condition outside the clinic, where wearing off and intrinsic and extrinsic environments impact performance, thus helping to assess functional status and fall risk. The underlying biological mechanism of gait disturbances and falls is rather complex and still to be fully defined. However, high variability and step-to-step fluctuations as extracted from body worn sensors may represent central neuronal rhythm deficits. Impaired internal rhythmicity (“biological clock”) is associated with inconsistency of the gait, leading to postural control impairment and consequentially disequilibrium and falls.

The present results also illustrate that activity monitors and step counters that only estimate the amount of activity apparently do not adequately capture fall risk in PD. The quantity of activity may be important for other health benefits like cardiovascular fitness, but quality may be more important than quantity when it comes to fall risk in PD. Further, the current findings set the stage for the provision of objective measures that are not dependent exclusively on self-report or a test at a single time point. In theory, sensor-derived metrics can capture wearing off, the effects of motor response fluctuations and changes in fall risk in response to the many motor and cognitive challenges during daily living. They should, therefore, assist in the evaluation of disease progression, the benefits of therapeutic interventions and fall risk. In the future, it may also be helpful to apply mediation and other types of analyses to further evaluate the relationship between the different measures. In the spirit of mHealth [13], [14], the routine clinical exam can be markedly augmented using continuously worn, body-fixed sensors that provide objective details about the patient's activity and fall risk.

## Note

The long-term recordings and the clinical data on which the present analyses were made will be available at www.physionet.org, the National Institutes of Health-sponsored *Research Resource for Complex Physiologic Signals*.

## Supporting Information

Table S1 **Correlations among the sensor derived measures.**
(DOC)

Table S2 **Descriptive properties and distributions among the sensor derived measures.**
(DOC)
