# Supplementary material for: Objective Assessment of Fall Risk in Parkinson's Disease Using a Body-Fixed Sensor Worn for 3 Days
Source: PLoS One. 2014 May 6;9(5):e96675. doi: 10.1371/journal.pone.0096675 (PMC4011791; doi:10.1371/journal.pone.0096675)
Supplement: Table S1 — Correlations among the sensor derived measures. (DOC) [file pone.0096675.s001.doc]

| **Table S1:** Correlations among the 3 day sensor-derived measures | | | | | | | | |
| --- | --- | --- | --- | --- | --- | --- | --- | --- |
| PCI | Harmonic Ratio | | | Stride Regularity [g^2] | | | **Axis** | **Measure** |
|  | ML | AP | **V** | ML | AP | **V** |  | Axis |
| -0.553 (<0.0001) | -0.224 (0.020) | 0.382 (<0.0001) | 0.276 (0.004) | .212 (0.028) | .422 (<0.0001) | .289 (0.0026) | Total percent of overall activity duration [%] | |
| -0.385 (<0.0001) | -0.143 (0.141) | 0.209 (0.031) | 0.223 (0.022) | 0.290 (0.002) | 0.385 (<0.0001) | 0.222 (0.022) | Median activity Bout duration [sec] | |
| -0.468 (<0.0001) | -0.290 (0.002) | 0.323 (0.001) | 0.137 (0.160) | 0.177 (0.069) | 0.175 (0.071) | 0.213 (0.028) | Cadence [steps/minute] | |
| -0.362 (0.0001) | -0.022 (0.820) | 0.401 (<0.0001) | 0.562 (<0.0001) | -0.017 (0.86) | .283 (0.003) | .763 (<0.0001) | V | Amplitude of dominant frequency [prs] |
| -0.323 (0.001) | -0.181 (0.062) | 0.472 (<0.0001) | 0.384 (<0.0001) | 0.097 0.322 | 0.596 (<0.0001) | 0.388 (<0.0001) | AP |
| 0.398 (<0.0001) | 0.074 (0.449) | -0.337 (0.0003) | -0.190 (0.051) | 0.082 (0.403) | -0.209 (0.031) | -0.263 (0.006) | ML |
| 0.547 (<0.0001) | 0.336 (<0.0001) | -0.491 (<0.0001) | -0.779 (<0.0001) | -0.323 (0.001) | -0.449 (<0.0001) | -0.851 (<0.0001) | V | Width of dominant frequency [Hz] |
| 0.820 (<0.0001) | 0.450 (<0.0001) | -0.807 (<0.0001) | -0.526 (<0.0001) | -0.278 (0.004) | -0.673 (<0.0001) | -0.55 (<0.0001) | AP |
| 0.336 (0.0003) | 0.356 (0.0001) | -0.196 (0.043) | -0.113 (0.250) | -0.430 (<0.0001) | -0.191 (0.048) | -0.200 (0.040) | ML |
