# Supplementary material for: Objective Assessment of Fall Risk in Parkinson's Disease Using a Body-Fixed Sensor Worn for 3 Days
Source: PLoS One. 2014 May 6;9(5):e96675. doi: 10.1371/journal.pone.0096675 (PMC4011791; doi:10.1371/journal.pone.0096675)
Supplement: Table S2 — Descriptive properties and distributions among the sensor derived measures. (DOC) [file pone.0096675.s002.doc]

| **Table S2:** Descriptive properties of the sensor derived measures | | | |
| --- | --- | --- | --- |
| **range** | **mean**± **sd** | **Measure** | |
| **Quantity measures (Activity count)** | | | |
| 1-117 | 31.07 ± 22.54 | Total Number of activity Bouts [#] a | |
| 0.023-10.84 | 2.22 ± 1.97 | Total percent of activity duration [%] | |
| 94.61-56435.77 | 10185.32±9569.82 | Total Number of steps for 3-days [#]  a | |
| 63.0-290.5 | 113.63±34.32 | Median activity Bout duration [sec] | |
| 9.26-564.86 | 195.71 ± 63.20 | Median Number of steps for Bout [#] | |
| 43.22-133.33 | 106.62±12.99 | Cadence [steps/minute] | |
| **Quality measures** | | | |
| 0.074-1.109 | 0.633±0.193 | V | Amplitude of dominant frequency [psd] |
| 0.088-0.926 | 0.583±0.149 | AP |
| 0.011-0.777 | 0.181±0.153 | ML |
| 0.634-1.452 | 0.745±0.140 | V | Width of dominant frequency [Hz] |
| 0.647-1.434 | 0.741±0.128 | AP |
| 0.531-1.514 | 0.935±0.145 | ML |
| 1.156-3.955 | 2.155±0.534 | V | Harmonic Ratio |
| 0.968-3.464 | 1.997±0.499 | AP |
| 0.312-1.121 | 0.617±0.147 | ML |
| 0.119-0.912 | 0.525±0.140 | V | Stride Regularity [g^2] |
| 0.264-0.822 | 0.533±0.103 | AP |
| 0.094-0.806 | 0.376±0.126 | ML |
| 1.777-49.317 | 7.293±8.413 |  | PCI |

These measures were calculated only from activity bouts ≥1min, from the AP axis a. Measures which were not normalized to the entire recording duration or activity duration.
